# Birth preparedness and complication readiness practice and associated factors among pregnant women in Central Ethiopia, 2021: A cross-sectional study

**DOI:** 10.1371/journal.pone.0276496

**Published:** 2022-10-27

**Authors:** Derara Girma, Addisu Waleligne, Hiwot Dejene

**Affiliations:** Public Health Department, College of Health Sciences, Salale University, Fitche, Oromia Regional State, Central Ethiopia; Sabzevar University of Medical Sciences, ISLAMIC REPUBLIC OF IRAN

## Abstract

**Background:**

Birth preparedness and complication readiness (BP/CR) is an intervention designated by the World Health Organization (WHO) as an essential element of the antenatal (ANC) package with a concept of a global strategy to reduce maternal mortality. In Ethiopia, the proportion of pregnant women preparing for birth and related complications has remained low. Whereas, the need for additional study is indicated to add more evidence to the country’s efforts to end preventable maternal death.

**Methods:**

A facility-based cross-sectional study was conducted from March 01 to May 01, 2021. A systematic random sampling technique was applied to recruit 422 pregnant women. Bivariable and multivariable binary logistic regression was fitted to identify factors associated with BP/CR practice. Variables with a p-value ≤ 0.25 on the bivariable analysis were included in multivariable analysis. Adjusted odds ratios (AOR) with the respective 95% confidence interval (CI) and a p-value <0.05 was used to set statistically significant variables in the multivariable analysis.

**Results:**

A total of 414 pregnant women have participated in the study. The overall BP/CR practice level was 44.9% (95% CI: 40.1, 49.7). Preconception care utilization (PCC) (AOR = 2.31; 95% CI:1.38–3.86), urban residents (AOR = 2.00; 95% CI:1.21–3.31), knowledge of BP/CR (AOR = 2.29; 95% CI:1.27–3.47), knowledge of danger signs during pregnancy (AOR = 2.05; 95% CI:1.21–3.47), knowledge of danger signs in newborns (AOR = 2.06; 95% CI:1.21–3.47), starting ANC visits in the 1^st^ and 2^nd^ trimester (AOR = 2.52; 95% CI:1.40–4.52), number of ANC visit ≥ three (AOR = 1.66; 95% CI;1.01–2.74), knowing Expected Date of Delivery (EDD) (AOR = 3.71; 95% CI:2.01–6.82), and joint decision-making on obstetric services (AOR = 3.51; 95% CI;1.99–6.20) were factors significantly associated with BP/CR practice.

**Conclusion:**

Based on the WHO standard, this study revealed a low level of BP/CR practice among pregnant women, with only less than half of women adequately prepared for childbirth and its complications. Moreover, it has been shown that BP/CR practice is influenced by socio-economic, maternal knowledge, and health service-related factors. Therefore, improving the status of BP/CR practice by expanding awareness creation opportunities, strengthening PCC and early ANC initiation by improving pregnant women’s understanding, and promoting joint decision-making on obstetric services are recommended.

## Introduction

Maternal health refers to the health of women during pregnancy, childbirth, and the postpartum period [1]. However, in 2017, nearly 810 women died from preventable causes related to pregnancy and childbirth daily. The majority of these deaths (94%) occurred in low-resource settings, with Sub-Saharan Africa (SSA) alone accounting roughly for two-thirds (66.4%) of maternal deaths [2]. Particularly, Ethiopia is listed as one of the 15 countries having the highest maternal mortality ratio (MMR) [2,3].

Birth Preparedness and Complication Readiness (BP/CR) is an intervention included by the World Health Organization (WHO) as an essential element of the Antenatal Care (ANC) package. It is a matrix of shared responsibilities involving the pregnant women/family, community, hospital facility/provider, and policymakers [4], also it is planning for normal birth and anticipating the actions needed in case of an emergency [5]. Whereas, Preconception Care (PCC) is the provision of biomedical, behavioral, and social health interventions to women and couples prior to conception. It aims to improve factors that contribute to poor maternal and child health outcomes through counseling, prevention, and management [6]. Despite their importance, the low-level practicability of these programs was identified as one of the major factors contributing to maternal mortality [4].

Furthermore, BP/CR has been linked to reduced maternal and neonatal mortality [7]. Similarly, according to WHO standards for maternal and newborn care, all pregnant women are required to have a written BP/CR plan [8]. However, the level of BP/CR in developing countries was found to be low, ranging from 12% in Bangladesh to 58.2% in Tanzania [5,9–14]. The situation is similar in Ethiopia, where the level of BP/CR practice ranged from 16.5%-57.7% [15–23], with a pooled estimate of only 25% of the pregnant women were prepared for birth and its complications [24].

Moreover, in previous studies so far, factors such as maternal education, number of ANC visits, knowledge of key danger signs [14], primigravida [10], history of obstetric complications, residence place, mother’s employment status [21], media exposures [25], wealth index, marital status [11], husbands educational status [19], women age, partners and/or family counseling [20], history of stillbirth [23], knowledge of obstetric complications [15] were significantly associated with BP/CR. Besides, traditional attitudes, resource shortages, financial limits, and misalignments between offered and expected maternity care services were also recognized as major implementation roadblocks of BP/CR [26].

Conversely, despite the fact that maternal health service utilization is regarded as the most important task in addressing maternal and child health (MCH) issues, significant disparities in service utilization exist both between and within Ethiopian regions [27]. Unexpectedly, the increasing trends of maternal health service utilization inequities were observed from the year 2000 to 2016 in the country [28]. Particularly, in central Ethiopia, a low rate of full ANC visits (only 33.7%) and a high rate of home deliveries (38.4%) were reported [29]. Additionally, during the COVID-19 pandemic, the area’s comprehensive maternal health service utilization rate (ANC visits based on gestational age, institutional delivery, and PNC (postnatal care) visits) was 64.8% [30]. Consequently, maternal health service utilization was significantly associated with BP/CR, implying the importance of BP/CR improvement in this context [31].

Despite the predicament of the problems, BP/CR is a devised strategy to reduce maternal and neonatal mortality globally [5,8], with a goal of less than 70 maternal deaths per 100,000 live births (LBs) [32] and 12 or fewer neonatal deaths per 1,000 LBs by 2030 respectively [33]. Specifically, Ethiopia has made significant progress in reducing MMR and has adopted a number of high-impact measures to improve maternal and newborn health services in recent years [34]. However, both maternal and newborn mortalities continue to be high. Maternal mortality has decreased slightly, from 871 deaths per 100,000 LBs in 2000 to 401 in 2017. Neonatal mortality has also decreased slightly, from 39 deaths per 1,000 LBs in 2000 to 33 in 2019 [35]. As a result, the need for additional studies measuring BP/CR levels was suggested in order to bring more evidence to the efforts of ending preventable maternal death in Ethiopia [20,22,36].

Furthermore, socio-economic and demographic variables could influence BP/CR practice levels among pregnant women, necessitating more research across various segments of pregnant women [24,26,37,38]. Additionally, although there were few prior studies in other regions [15–23] they failed in identifying proximate factors. Besides, there has been no prior study in the study area.

Overall, given the aforementioned rationale, this study aimed at assessing BP/CR practice and associated factors among pregnant women in Central Ethiopia, 2021. As a result, this finding will aid in policymaking and developing/strengthening evidence-based strategies to increase BP/CR practice, lowering maternal morbidity and mortality.

## Methods

### Study design, settings, and population

A facility-based cross-sectional study design was carried out at all North Shewa Zone public hospitals of Oromia regional state, central Ethiopia from March 01 to May 01, 2021. Fitche General Hospital, Kuyu general hospital, Muketuri Primary Hospital, Gundo Meskel Primary Hospital, and Sheno Primary Hospital were all public hospitals found in the zone and included in this study. Pregnant women living in the catchment areas routinely receive ANC at these public hospitals. And, all pregnant women who are attending ANC follow-up service in these hospitals during the study period were eligible for the study.

### Sample size and sampling method

**Fig 1:**
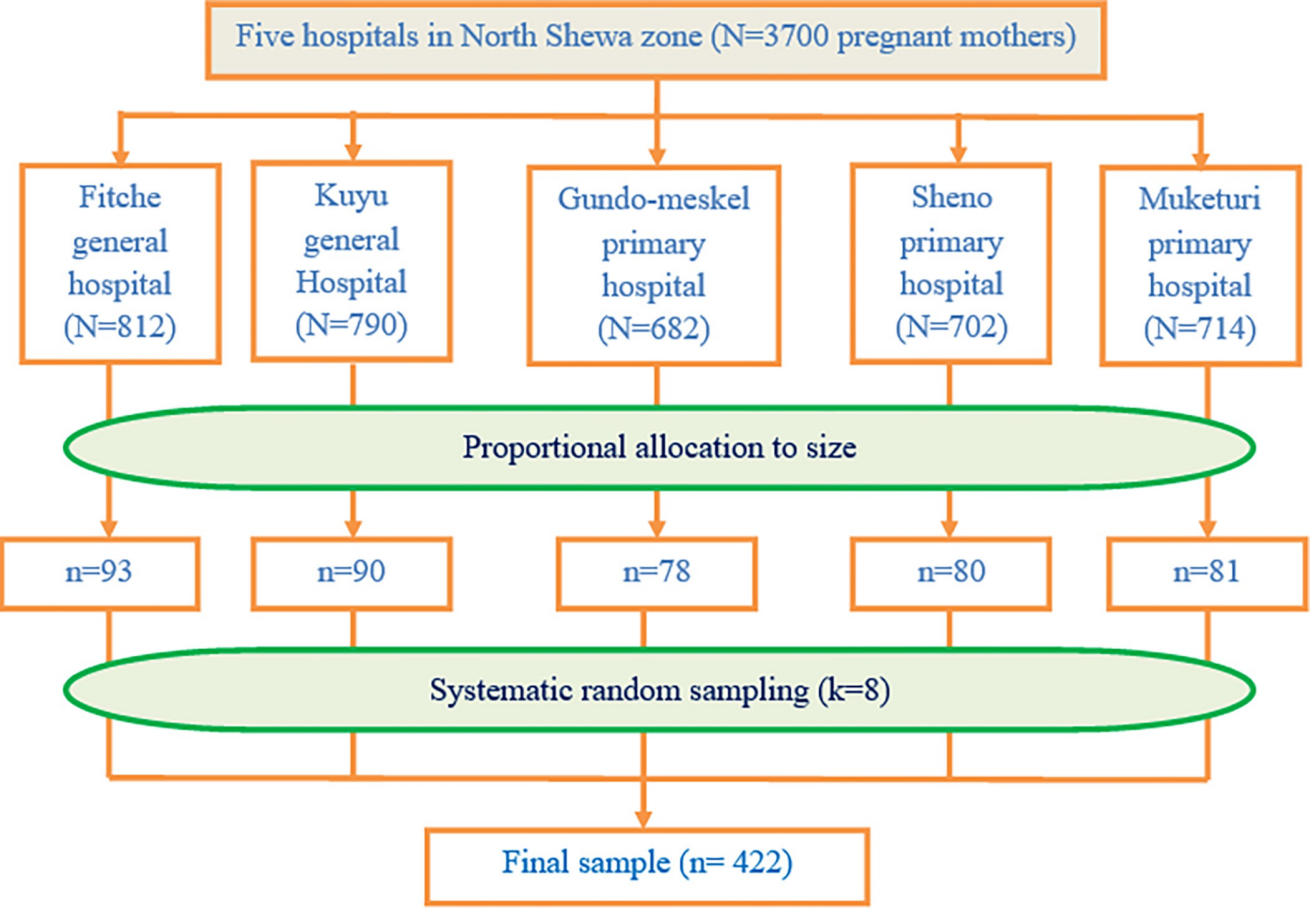
Schematic presentation of sampling procedure.

The sample size was determined by single population proportion formula using Epi Info stat calc. version 7.2, based on the following assumptions. 48.5% proportion of BP/CR practice [20], confidence level of 95%, margin of error (d) of 5%, and 10% non-response rate. Therefore, the final sample size obtained was 422. Then, the sample size was distributed using proportional allocation to size (PAS) to each hospital in the zone. Finally, a systematic random sampling technique was applied to recruit study participants (Fig 1).

### Measurements

Pregnant women were asked to mention the components of BP/CR simultaneously that were adapted from a survey tool developed by JHPIEGO maternal and neonatal health program [4]. Pregnant women were considered prepared for birth and its complication when they practiced or applied at least six items among twelve components of BP/CR [18]. The items of BP/CR have acceptable internal consistency in the current study (Cronbach-alpha = 0.85). The utilization of PCC was measured by the World Health Organization package of PCC services. A pregnant woman who utilized at least one component of the WHO package of PCC services for the current pregnancy was considered as PCC utilizer. The tool items had strong internal consistency (Cronbach-alpha = 0.76) in Ethiopia [39]. The items of PCC have acceptable internal consistency in the current study (Cronbach-alpha = 0.82). The wealth index was measured by a simplified and updated Ethiopian wealth index equity tool. The tool contains 15 simplified household assets measuring questions. The tool has an 84.2% agreement and 0.755 kappa statistics with the full Ethiopian Demographic Health Survey (EDHS,2016) wealth index measurement tool. Accordingly, the wealth index of the household was classified into five quintiles (quintile1-5). Those in the 1st and 2nd quintiles were classified as poorest (40%), those in the 3rd quintile were middle (20%), and those in the 4th and 5th quintiles were richest (40%) [40]. The principal component analysis was done to quantify the wealth index. The tool items were encompassed in factor analysis to explore the underlying component and to reduce the number of items and decide a number of principal components to be retained based on the eigenvalue. Factors with an eigenvalue greater than one were considered in the consequent analysis to characterize the variables using the varimax rotation method. Of the total fifteen (15) wealth index items included in the principal component analysis, factor analysis was considered to be suitable with the 10 items. For the final stage, it was observed that all items were correlated at ≥0.3 with at least one other item, but at <0.9 suggesting reasonable factorability. Secondly, the Kaiser-Meyer-Olkin measure of sampling adequacy was 0.73 and Bartlett’s test of sphericity was significant (p <0.001). The diagonals of the anti-image correlation matrix and communalities were also all >0.5. Overall, principal component analysis of the remaining 10 items has produced four components explaining the total variance of 75.5%. The inter-item consistency of all factor loadings of each component was computed and it generated a Cronbach alpha of 0.75. Using the regression factor score, after checking for the outlier and classifying the value, binary logistic regression analysis was performed and the effect of wealth index on the BP/CR was computed.

### Operational definitions

**BP/CR practice:** a pregnant woman was considered prepared for birth and its complication when she practiced or applied at least six items among twelve components of birth preparedness and complication readiness [18].

**PCC utilization:** a pregnant woman who utilized at least one component of the WHO package of PCC services for the current pregnancy was considered as PCC utilizer [39].

**BP/CR knowledge:** a pregnant woman was considered knowledgeable for birth preparedness and complication readiness when she spontaneously mentioned at least six items of birth preparedness and complication readiness [18].

**Knowledge of key danger signs during pregnancy:** a pregnant woman was considered knowledgeable about the danger signs of pregnancy when she mentioned at least three key danger signs of pregnancy [18].

**Knowledge of key danger signs during labor and delivery:** a pregnant woman was considered knowledgeable about the danger signs of labor and delivery when she mentioned at least three key danger signs of labor and delivery [18].

**Knowledge of key danger signs during the postpartum period:** a pregnant woman was considered knowledgeable about the danger signs of the postpartum period when she mentioned at least three key danger signs of postpartum [18].

**Knowledge of key danger signs of the newborn**: a pregnant woman was considered knowledgeable about the danger signs of the newborn if she mentioned four key danger signs of the newborn neonate [38].

### Data quality control

To ensure measurement validity, a standard questionnaire of BP/CR tools and indicators for maternal and newborn health was used. Also, all questionnaires were translated to the local languages, (Afan Oromo) by two independent bilingual translators and back-translated to English to guarantee consistency. The two-day training was given to the data collectors. The supervisors were also trained on how to monitor the data collection procedures. The instrument was pretested on 10% of the sample size at Chancho hospital and necessary modifications were made after the pretest. As well, a reliability test was done and tools with Cronbach-alpha >0.7 were used in the actual data collection.

### Data processing and analysis

Data were entered into Epi Data Entry client version 4.6.0.5 and exported into STATA-14 for analysis. Data exploration was carried out to assess completeness and descriptive statistics were used to describe the study participants’ data based on its nature. Bivariable analysis and crude odds ratio (COR) with a 95% confidence interval (CI) was used to realize the association between an independent variable and the outcome variable using binary logistic regression. All independent variables with a p-value ≤0.25 in the bivariable analysis were simultaneously included in multivariable analysis to control for the effect of potentially confounding factors. Multiple logistic regression was performed using a backward stepwise method to identify factors independently associated with the outcome variable. The adjusted odds ratio (AOR) with a 95% confidence interval (CI) was computed to measure the strength of the association. Finally, a p-value <0.05 was declared a statistically significant variable. A multi-collinearity diagnostic test was conducted to check for collinearity among the independent variables using VIF with the threshold of 10 and there was no multicollinearity detected between the variables (the maximum VIF was 1.35). As well the model fitness was checked using the Hosmer and Lemeshow goodness-of-fit model and the model was fitted (p-value = 0.245).

### Ethical consideration

Ethical clearance was obtained from the ethical review committee of Salale University. The permission letter was obtained from the administrative office of the North Shewa zone Health Bureau and was given to all public hospitals. Written informed consent was obtained from each study participant. Confidentiality was assured by data collectors.

## Results

### Sociodemographic characteristics

A total of 414 pregnant women have participated in the study. The median (IQR) age of the women was 26 (9) years with a minimum and maximum age of 18 and 42 years old respectively. More than half, 247 (59.66%) were urban residents and 82 (19.81%) were in medium wealth index level. Regarding family size, half of the study participants, 206 (49.76%) have 4–6 family sizes (Table 1).

**Table 1:** Socio-demographic characteristics of pregnant women attending antenatal care service at North Shewa zone public hospitals, Oromia region, central Ethiopia, 2021 (n = 414).

Variable	Category	Frequency (percentage)
**Age**	≤ 24	149 (35.99)
	25–34	172 (41.55)
	≥35	93 (22.46)
**Residence**	Urban	247 (59.66)
	Rural	167 (40.34)
**Marital status**	Married	398 (96.14)
	Divorced	7 (1.69)
	Others *	9 (2.18)
**Educational status**	No formal education	32 (7.73)
	Primary (1–8 grade)	138 (33.33)
	Secondary (9–12 grade)	116 (28.02)
	Diploma and above	128 (3.92)
**Wealth index level**	Poorest	166 (40.10)
	Medium	82 (19.81)
	Richest	166 (40.10)
**Occupation**	Employee	123 (29.71)
	Merchant	81 (19.57)
	Farmer	52 (12.56)
	Housewife	113 (27.29)
	Others ^**Ɨ**^	45 (10.87)
**Family size**	1–3	164 (39.61)
	4–6	206 (49.76)
	≥7	44 (10.63)
**Distance from home to hospital**	≤5km	245 (59.18)
	>5km	169 (40.82)
**Husband education level**	No formal education	34 (8.54)
	Primary (1–8 grade)	75 (18.84)
	Secondary (9–12 grade)	96 (24.12)
	Diploma and above	193 (48.49)
**Decision maker for obstetric service seeking**	Joint wife and husband	280 (67.63)
	Others^**+**^	134 (32.37)
**Gravidity**	Primigravida (1)	151 (36.47)
	multigravida (2–4)	246 (59.42)
	Grand-multigravida (≥5)	17 (4.11)
**Current gestational age**	First Trimester	57(13.77)
	Second Trimester	164 (39.61)
	Third Trimester	193 (46.62)

*Single, Widowed; **Ɨ** Student, Daily laborer **+** self, husband relatives, wife relatives.

### Knowledge of BP/CR, key danger signs during pregnancy, labor, the postpartum and newborn period

Out of the total participants, more than three-fourths, 318 (76.81%) had a knowledge of BP/CR i.e., the pregnant women spontaneously mentioned at least six and above of the BP/CR knowledge question items. Besides, this study showed that 390 (94.20%), 345 (83.33%), and 321 (77.54%) of the pregnant women were able to mention saving money, place of delivery, and preparing clean clothes as items of BP/CR respectively (Table 2).

**Table 2:** BP/CR knowledge items among pregnant women attending antenatal care service at North Shewa zone public hospitals, Oromia region, central Ethiopia, 2021 (n = 414).

Birth preparedness and complication readiness items	Frequency (percentage)
Identify the place of delivery	345 (83.33)
Identify skilled provider	292 (70.53)
Save money	390 (94.20)
Identify means of emergency transport	238 (57.49)
Arrange a blood donor for emergency	143 (34.54)
Identify emergency obstetric signs	154 (37.20)
Identify health institution with 24 hours emergency obstetric care	220 (53.14)
Prepare clean clothes & other materials	321 (77.54)
Arrange for an emergency fund	207 (50.00)
Make a plan for communication means	170 (41.06)
Identify support people to help	171 (41.30)
Identify the importance of seeking care without delay	185 (44.69)

**Fig 2:**
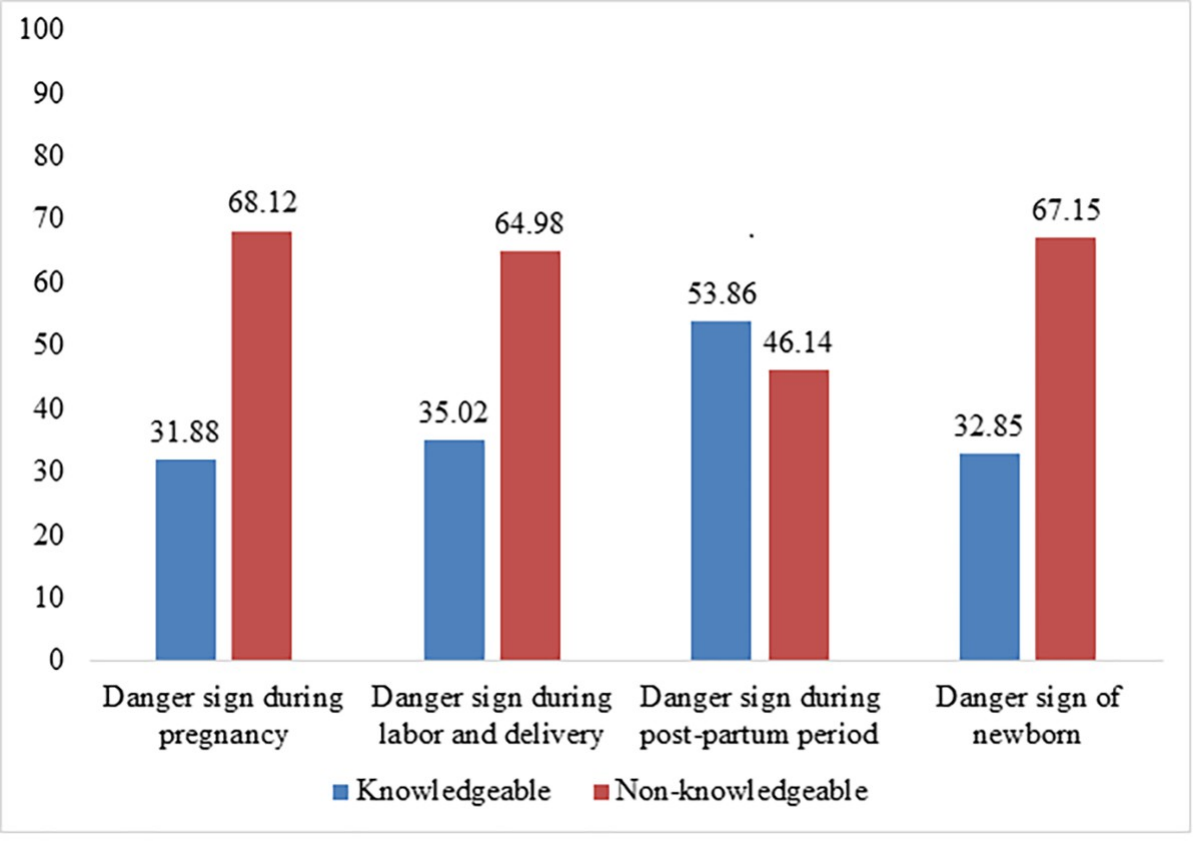
Knowledge of danger signs among pregnant women.

Regarding knowledge of danger signs during pregnancy, nearly one-third of the study participants, 132 (31.88%) are knowledgeable. Moreover, more than a third, 145 (35.02%) have knowledge of danger signs during labor and delivery, as well nearly more than half, 223 (53.86%) have knowledge of danger signs that can occur during the post-partum period. Additionally, one-third, 136 (32.85%) of the study participants have a knowledge of danger signs that can occur in newborns (Fig 2). Concerning the knowledge measuring items comprised in the study; vaginal bleeding 191 (46.14%) was stated as a major danger sign occurring during pregnancy, while prolonged labor (>12 hours) 252 (60.87%) was mentioned as a top danger sign that can occur during labor and delivery. Also, a foul-smelling vaginal discharge, 199 (48.07) and poor sucking or feeding, 222 (53.62) were stated as the main danger signs that can occur during the postpartum period and in newborns respectively (Table 3).

**Table 3:** Items used to measure knowledge of danger signs during pregnancy, labor and delivery, postpartum, and in newborns among pregnant women attending antenatal care service at North Shewa zone public hospitals, Oromia region, central Ethiopia, 2021 (n = 414).

Danger signs	Frequency (percentage)
**During pregnancy**	
Vaginal bleeding	191 (46.14)
Swollen hands/face	91 (21.98)
Blurred vision	157 (37.92)
Severe headache	120 (28.99)
Convulsion	109 (26.33)
Severe abdominal pain	157 (37.92)
**During labor and delivery**	
Severe Vaginal bleeding	229 (55.31)
Prolonged labor (>12 hour)	252 (60.87)
Convulsion	100 (24.15)
Retained placenta	154 (37.20)
Severe headache	48 (11.59)
Hand feet cord face appears first	26 (6.28)
**During postpartum period**	
Severe Vaginal bleeding	180 (43.48)
foul-smelling vaginal discharge	199 (48.07)
Convulsion	120 (28.99)
High fever	176 (42.51)
Swollen hands and face	136 (32.85)
Severe headache	153 (36.96)
Blurred vision	130 (31.40)
**Newborn**	
Difficult or fast breathing	172 (41.55)
Yellow skin or eye color	153 (36.96)
Poor sucking or feeding	222 (53.62)
Bleeding, or discharge from around the umbilical cord	166 (40.10)
Baby very small	43 (10.39)
Convulsion/spasm/rigidity	125 (30.19)
Lethargy/unconsciousness	119 (28.74)

### Preconception Care (PCC) Utilization and other obstetrics factors

**Fig 3:**
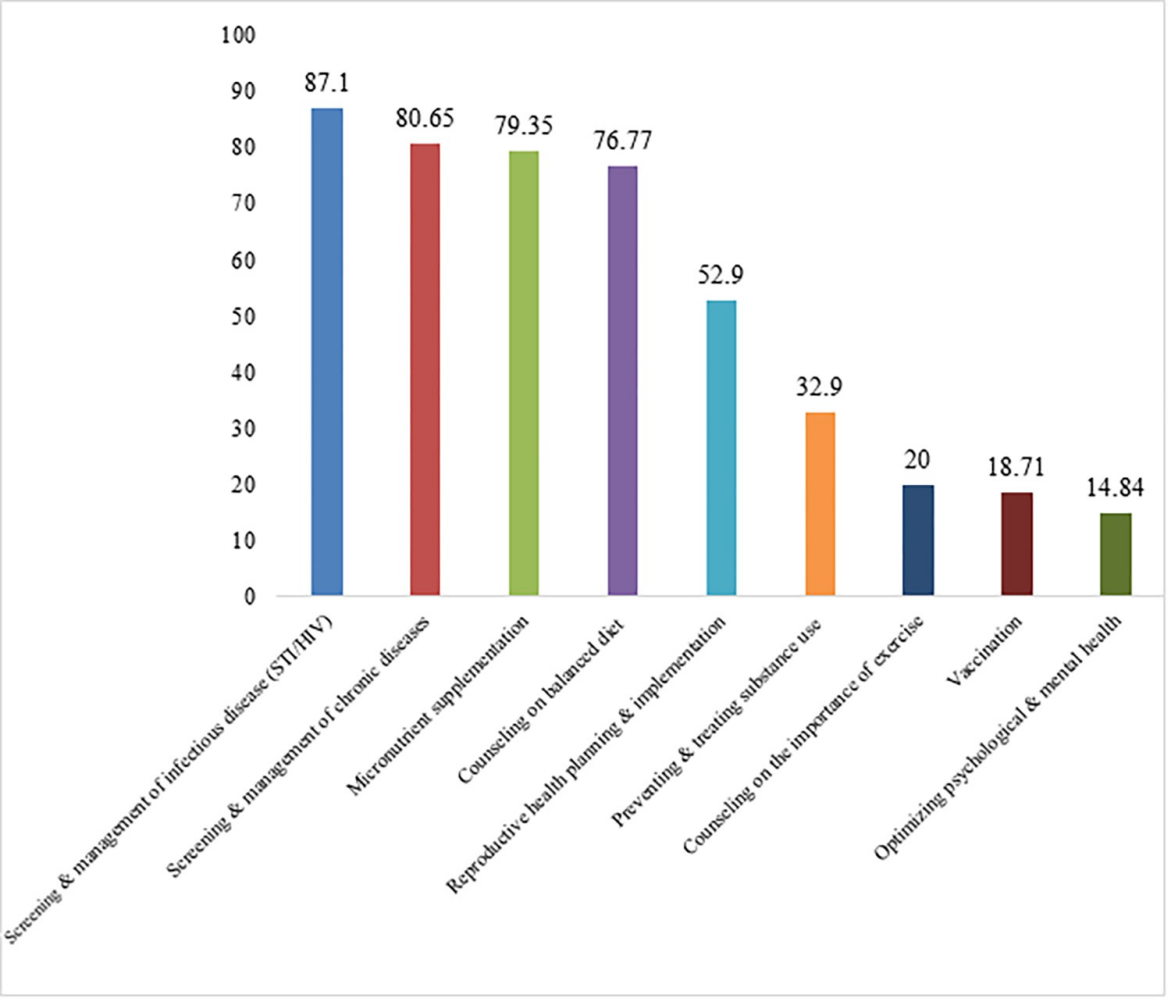
Percentage of WHO components of PCC utilization among pregnant women.

According to WHO recommendations, more than a third of pregnant women, 155 (37.44%), used at least one component of the PCC package before the current pregnancy. Of the recommended PCC packages, the most commonly utilized were; screening and management of infectious diseases (STI/HIV) 135 (87.10%), screening and management of chronic diseases 125 (80.65%), and micronutrient supplementation (i.e., iron, folic acid) 123 (79.35%) (Fig 3).

### Level of BP/CR practice

The overall BP/CR practice level among pregnant women was 44.9% (95% CI: 40.1, 49.7). Accordingly, among the arrangements made ahead of the current pregnancy; 388 (93.72%) saved money for an emergency, 321 (77.54%) identify the place of delivery, and 244 (58.94%) prepared clean clothes and other materials (Table 4).

**Table 4:** Arrangement items for BP/CR plan among pregnant women attending antenatal care service at North Shewa zone public hospitals, Oromia region, central Ethiopia, 2021 (n = 414).

Birth preparedness and complication readiness items	Frequency (percentage)
Identify the place of delivery	321 (77.54)
Identify skilled provider	221 (53.38)
Save money	388 (93.72)
Identify means of emergency transport	153 (36.96)
Arrange a blood donor for emergency	107 (25.85)
Identify emergency obstetric signs	102 (24.64)
Identify health institution with 24 hours emergency obstetric care	152 (36.71)
Prepare clean clothes & other materials	244 (58.94)
Arrange for an emergency fund	124 (29.95)
Make a plan for communication means	105 (25.36)
Identify support people to help	121 (29.23)
Identify the importance of seeking care without delay	79 (19.08)

### Factors associated with BP/CR practice

Out of the factors included in the model, residence, PCC utilization, gestational stage at 1^st^ ANC, number of ANC visits, knowing the EDD, decision-maker on obstetric service seeking, knowledge of BP/CR, danger signs during pregnancy, and newborn were significantly associated with BP/CR. Accordingly, the odds of BP/CR practice among urban residents were two times higher than the odds of BP/CR practice among rural residents (AOR = 2.00; 95%CI:1.21–3.31). The odds of practicing BP/CR were more than twice greater among pregnant women who had knowledge of BP/CR than their counterparts (AOR = 2.29; 95%CI:1.27–3.47). The practice of BP/CR was two times more common among pregnant women who had knowledge of danger signs during pregnancy than their counterparts (AOR = 2.05; 95%CI:1.21–3.47). Pregnant women who had knowledge of danger signs in newborns were two times more likely to practice BP/CR than their counterparts (AOR = 2.06; 95%CI:1.21–3.47). Furthermore, the odds of practicing BP/CR were more than twice greater among pregnant women who had utilized PCC than those who had not utilized PCC (AOR = 2.31; 95%CI:1.38–3.86). Besides, pregnant women who had started ANC visits during the 1st and 2nd trimesters were more than two times more likely to practice BP/CR than those who had started during the 3rd trimester (AOR = 2.52; 95%CI:1.40–4.52). The odds of BP/CR practice among pregnant women who had a number of ANC visits ≥3 were almost two times higher than the odds of BP/CR practice among pregnant women who had number of ANC visits ≤2 (AOR = 1.66; 95%CI;1.01–2.74). Pregnant women who did know their EDD were almost four times more likely to practice BP/CR than those who did not know their EDD (AOR = 3.71; 95%CI:2.01–6.82). Similarly, pregnant women who decide on obstetric service seeking with their husbands were more than three times more likely to practice BP/CR than those whose decision is given by others (AOR = 3.51; 95%CI;1.99–6.20) (Table 5).

**Table 5:** Factors associated with BP/CR among pregnant women attending antenatal care service at North Shewa zone public hospitals, Oromia region, central Ethiopia, 2021 (n = 414).

Variable	Category	Birth preparedness and complication readiness		COR (95%CI)	AOR (95%CI)
		Yes (%)	No (%)		
Residence	Urban	135 (54.66)	112 (45.34)	**2.74 (1.81–4.15)**	**2.00(1.21–3.31)** *
	Rural	51 (30.54)	116 (69.46)	1	1
Educational status	Diploma and above	76 (59.38)	52 (40.63)	2.34 (1.53–3.58)	1.37 (0.80–2.34)
	≤12 grade	110 (38.46)	176 (61.54)	1	1
Family size	1–3	95 (57.93)	69 (42.07)	4.13 (1.95–8.74)	0.69 (0.24–1.96)
	4–6	80 (38.83)	126 (61.17)	1.90 (0.91–3.98)	0.27 (0.09–1.77)
	≥7	11 (25.00)	33 (75.00)	1	1
Knowledge of BP/CR	Knowledgeable	162 (50.94)	156 (49.06)	**3.12 (1.87–5.19)**	**2.29 (1.27–3.47)** *
	Unknowledgeable	24 (25.00)	72 (75.00)	1	1
Knowledge of danger signs during labor and delivery	Knowledgeable	83 (57.24)	62 (42.76)	2.16 (1.43–3.25)	1.37 (0.82–2.30)
	Unknowledgeable	103 (38.29)	166 (61.71)	1	1
Knowledge of danger signs during pregnancy	Knowledgeable	87 (65.91)	45 (34.09)	**3.57 (2.31–5.52)**	**2.05 (1.21–3.47)** *
	Unknowledgeable	99 (35.11)	183 (64.89)	1	1
Knowledge of danger signs during the postpartum period	Knowledgeable	114 (51.12)	109 (48.88)	1.73 (1.17–2.56)	1.26 (0.75–2.11)
	Unknowledgeable	72 (37.70)	119 (62.30)	1	1
Knowledge of danger signs in newborn	Knowledgeable	81 (59.56)	55 (40.44)	**2.43 (1.59–3.69)**	**2.06 (1.21–3.47)** *
	Unknowledgeable	105 (37.77)	173 (62.23)	1	1
Preconception care utilization	Yes	106 (68.39)	49 (31.61)	**4.84 (3.15–7.43)**	**2.31 (1.38–3.86)** *
	No	80 (30.89)	179 (69.11)	1	1
Current gestational age	Third trimester	78 (40.41)	115 (59.59)	1.35 (0.73–2.52)	0.53 (0.23–1.18)
	Second trimester	89 (54.27)	75 (45.73)	2.37 (1.26–4.46)	1.11 (0.49–2.52)
	First trimester	19 (33.33)	38 (66.67)	1	1
Gestational age at first ANC visit	1^st^ and 2^nd^ trimester	153 (49.35)	157 (50.65)	**2.09 (1.31–3.35)**	**2.52 (1.40–4.52)** *
	Third trimester	33 (31.73)	71 (68.27)	1	1
Number of ANC visit	≥3	116 (51.56)	109 (48.44)	**1.81 (1.22–2.68)**	**1.66 (1.01–2.74)** *
	1–2	70 (37.04)	119 (62.96)	1	1
Current pregnancy planned	Yes	171 (51.20)	163 (48.80)	4.55 (2.49–8.29)	1.38 (0.65–2.92)
	No	15 (18.75)	65 (81.25)	1	1
Know Expected Date of Delivery	Yes	164 (56.16)	128 (43.84)	**5.82 (3.47–9.76)**	**3.71 (2.01–6.82)** *
	No	22 (18.03)	100 (81.97)	1	1
The decision maker in service seeking	Jointly	159 (56.79)	121 (43.21)	**5.21 (3.21–8.45)**	**3.51 (1.99–6.20)** *
	Others ^**+**^	27 (20.15)	107 (79.85)	1	1

^+^Self, husband/ wife relatives; COR = Crude Odds Ratio; CI = Confidence Interval; AOR = Adjusted Odds Ratio; 1 = reference

* Significant at p-value <0.05.

## Discussion

This study assessed BP/CR and associated factors among pregnant women in central Ethiopia, in 2021. BP/CR interventions are suggested to increase the use of skilled birth care [37,41] and the timely use of facility care for obstetric and newborn complications [41]. As well, it is effective in reducing neonatal mortality in low-resource settings [7]. However, the practical indication of the need for preconception investigations, the existence of ignorance in postpartum women and newborns, and the lack of full BP/CR package implementation realize the necessity for alike studies in Ethiopia [42].

Accordingly, in this study, the overall BP/CR level among pregnant women was 44.9%. This finding was in line with the study done in Benchi-Maji zone, Ethiopia (42.3%) [43], Sodo town, Ethiopia (48.5%) [20], and Nigeria (40.3%) [44]. However, this finding was higher than the study done in Ghana (15%) [45] and Bangladesh (24.5%) [46]. This might be due to socio-demographic variation, application of different exclusion criteria, and study setting differences. Moreover, this result was also higher than the previous studies done in Ethiopia; such as in South Wollo (24.1%) [16], Agnuak zone (25.8%) [21], Adama (29.1%) [23], Goba woreda (29.9%) [17], Arba Minch Zuria Woreda (30%) [22], and Farta district (34%) [18]. This can due to a current study was conducted among hospital ANC attendants which can increase the possibility of BP/CR [20]. Furthermore, the knowledge of BP/CR in this study is much higher than in previous studies, which can increase the practice of BP/CR [38]. Additionally, the current quality of healthcare and availability of information about BP/CR is not the same as the previous years [43].

In contrast, the level of BP/CR in this study was lower than the study done in Uganda (53.9%) [25], Nigeria (81.5%) [47], Tanzania (95%) [48], Nepal (75.2%) [49], and Ethiopia (54.7%) [50]. The difference may attributable to the level of the study settings because those studies were only conducted at referral and teaching level hospitals. Additionally, lack of access to ANC as a result of COVID-19 pandemic-related restrictions may also reduce mothers’ visits to health facilities, resulting in a low BP/CR practice detection rate in the current study [51]. Furthermore, recent evidence revealed that the WHO-recommended BP/CR plan was all significantly affected by the COVID-19 pandemic [52].

In this study, urban residents practice more BP/CR than rural residents. This is supported by the studies from Benchi-Maji [43] and Farta district [18]. This might be due to dissimilarity in access to information, education, accessibility, and availability of maternal health services [18]. Having knowledge of BP/CR increases the practice of BP/CR among pregnant women in this study. This is comparable with the studies from Benchi-Maji [43], Farta district [18], and Nepal [49]. The inference of this finding could be once women become knowledgeable about BP/CR, they are expected to practice the components of BP/CR and ready to act upon it when it occurs [43]. Also, knowledge of danger signs during pregnancy and danger signs of the newborn were significantly associated with BP/CR practice. This is supported by studies from the Agnuak zone, Ethiopia [21], Tehulederie district, Ethiopia [53], Rwanda [10], Ghana [45], and Nepal [49]. This might be due to women who did know key obstetric-related danger signs and complications have a higher intention to seek care related to pregnancy and childbirth [21]. Furthermore, a lack of awareness of obstetric danger signs may result in a lack of preparedness for normal BP/CR in the event of obstetric complications that necessitate emergency care [45].

Pregnant women who had utilized PCC had an increased likelihood of BP/CR practice. This is due to PCC utilization will improve mothers, neonates, and children’s health outcomes by promoting BP/CR practice [54]. Moreover, because childbirth preparation counseling is one of the PCC components, its use will lead to increased BP/CR practice. Pregnant women who had started ANC visits during the 1^st^ and 2^nd^ trimesters and who had a number of ANC visits ≥3 times had a heightened chance for BP/CR practice. This is supported by a study from Agnuak zone, Ethiopia [21] and Tehulederie district, Ethiopia [53]. This is because ANC is more effective and results in pregnancy risk reduction when initiated earlier in the course of the pregnancy [4]. Also, there is better exposure to information regarding BP/CR in the frequent ANC visits [53].

Moreover, the greater chance of BP/CR among pregnant women who know their EDD in our study was similar to the report of another study conducted in India [55]. This could be because respondents’ knowledge of their due date will lead them to be well prepared in advance to avoid any complications. Also, those pregnant women who decide on obstetric service seeking jointly with their husbands were more likely to practice BP/CR. This is supported by a study from Nepal [49]. It is critical to actively involve the husband in maternal and newborn health. This engagement might also have cultural influences. It has also recognized the significance of advocating for policies and strategies that can increase men’s awareness and involvement in maternal care [56].

## Conclusion

According to WHO standards for maternal and newborn care, all pregnant women are expected to have a written BP/CR practice plan [8]. Based on this standard, this study revealed a low level of BP/CR practice among pregnant women, with only less than half of women adequately prepared for childbirth and its complications. This inadequacy in BP/CR practice could mean that majority of deliveries among pregnant women will not be attended by skilled birth attendants. With such an alarming figure, the likelihood of lowering Ethiopia’s currently high maternal mortality ratio appears unlikely, making the 2030 Sustainable Development Goals (SDGs) maternal mortality ratio target appear implausible. Moreover, factors such as PCC utilization, residence, knowledge of BP/CR, danger signs during pregnancy and danger signs in newborns, gestational stage at 1^st^ ANC, number of ANC visits, knowing the EDD, and joint decision making on obstetric service-seeking were significantly associated with BP/CR practice. Therefore, improving the status of BP/CR practice by expanding awareness creation opportunities, strengthening PCC and early ANC initiation by improving pregnant women’s understanding, and promoting joint decision-making on obstetric services are recommended.

### Limitation of the study

Being a cross-sectional study, made it impossible to draw causal inferences. Some self-reported responses will be subjected to social desirability bias.

## Supporting information

S1 File Minimal data set. (DTA)

S2 File Questionnaires. (DOCX)

## Abbreviations

ANC: Antenatal Care
BP/CR: Birth Preparedness and Complication Readiness
JHPIEGO: Johns Hopkins Program for International Education in Gynecology and Obstetrics
PCC: Preconception Care
WHO: World Health Organization
