# Supplementary material for: Birth preparedness and complication readiness practice and associated factors among pregnant women in Central Ethiopia, 2021: A cross-sectional study
Source: PLoS One. 2022 Oct 27;17(10):e0276496. doi: 10.1371/journal.pone.0276496 (PMC9612452; doi:10.1371/journal.pone.0276496)
Supplement: S2 File — (DOCX) [file pone.0276496.s002.docx]

| **Part-1: Socio-demographic characteristics of antenatal mothers** | | | |
| --- | --- | --- | --- |
| S. No | Variables | Options | Skip |
| 101 | Age (in a completed year) | __________ |  |
| 102 | Residence | 1. Urban 2. Rural |  |
| 103 | What is your marital status? | 1. Single 2. Married 3. Divorced 4. Widowed 5. Others (specify)__ |  |
| 104 | What is your religion? | 1. Orthodox 2. Protestant 3. Muslim 4 Others (specify)________ |  |
| 105 | What is your ethnicity? | 1. Oromo 2. Amhara 3. Gurage 4. Others (specify)__________ |  |
| 106 | What is your educational level? | 1. No formal education 2. Primary education (1-8 grade) 3. Secondary (9-12 grade) 4. Diploma and above |  |
| 107 | What is your husband educational level? | 1. Unable to read and write 2. Primary education (1-8 grade) 2. Secondary (9-12 grade) 4. Diploma and above 3. I do not know |  |
| 108 | What is your occupation? | 1. Employee 2. Merchant 3. Farmers 4. Housewife 2. Others (specify)________ |  |
| 109 | Your husband/partner occupation? | 1. Farmer 2. Employee 3. Merchant 4. Others (specify)________ |  |
| 110 | What is your family size (in number) | ____________________ |  |
| 111 | Who earn an income in family? | 1. Wife only 2. Husband only 3. Both 4. None |  |
| 112 | What is the distance from your home to health institution? (in kilometer) | ____________ |  |
| **Part-2: knowledge on birth BP& CR, key danger sign of pregnancy, labor, postpartum and newborn period.** | | | |
| 201 | Have you heard/counseled about BP/CR? | 1. Yes 2. No | If “no” go-to 203 |
| 202 | If your answer to **Q 201 is yes,** what is your Source of information of BP/CR? | 1. Your relative 2. Mass media 3. Health professionals 2. Your friend 5. Health extension workers 5. Others (specify) _____ |  |
| 203 | What do you know about components of preparation for birth and its complication readiness? **(possible to select more than one answer)** | 1. Identify place of delivery 2. Identify skilled provider 3. Save money 4. Identify means of emergency transport 5. Arrange a blood donor for emergency 6. Identify emergency obstetric signs 7. Identify health institution with 24 hours emergency obstetric care 8. Prepare clean clothes & other materials 9. Arrange for emergency fund 10. Make a plan for communication means 11. Identify support people to help 12. Identify the importance of seeking care without delay |  |
| 204 | When do you think obstetric danger signs can occur? **(possible to select more than one answer)** | 1. During pregnancy 2. During labor and delivery   3.During post-partum period 4. I do not know | If “I don’t know” go-to 210 |
| 205 | What types of obstetric danger signs occur during pregnancy? **(possible to select more than one answer)** | 1. Vaginal bleeding 2. Swollen hands/face 3. Blurred vision  4. Severe headache 5. Convulsion 6. Severe abdominal pain  7. Others (specify) ___________ |  |
| 206 | What types of Obstetric danger signs can occur during labour and deliver?  **(possible to select more than one answer)** | 1. Severe vaginal bleeding 2. Prolonged labor (>12huors)  3. Convulsion 4. Retained placenta 5. Severe headache  6. Hand, feet, cord or face appears first  7. Others (specify) ____________ |  |
| 207 | What types of danger signs can occur during post-partum period (in the first 2 days after birth)? **(possible to select more than one answer)** | 1. Severe vaginal bleeding 2. Foul-smelling vaginal discharge  3. Convulsion 4. High fever 5. Swollen hands/face  6. Severe headache 7. Blurred vision  8. Others (specify) ___________ |  |
| 208 | What types of danger signs can occur in the **first 7 days** after birth that could endanger the life of a newborn baby? **(possible to select more than one answer)** | 1. Difficult or fast breathing 2. Yellow skin/eye color  3. Poor sucking or feeding 4. Bleeding, or discharge from around the umbilical cord 5. Baby very small 6. Convulsions/spasms/rigidity  7. Lethargy/unconsciousness 8. Others (specify) ___________ |  |
| 209 | What are the basic cares that can be provided to a newborn baby after birth? **(multiple answer is possible)** | 1. Exclusive breastfeeding 2. Dry and wrap eyes 3. Cord care  4. Feeding food/Water 5. Others (specify) __________ |  |
| **Part-3: preconception care related factors** | | | |
| 301 | Have you visited health facilities before you got a current pregnancy to utilize pre-conception care? | 1. Yes 2. No | If “no go-to Q401 |
| 302 | If **“yes”** to **Q301**, what type of services did you get? **(multiple answer is possible)** | 1. Micronutrient supplementation 2. Vaccination 3. Screening and management of chronic diseases 4. Screening and management of infectious disease (STI/HIV) 5. Counseling on balanced diet 6. Reproductive health planning and implementation 7. Optimizing psychological and mental health 8. Preventing and treating substance use 9. Counseling on the importance of exercise |  |
| **Part-4: Obstetrics and antenatal related factors** | | | |
| 401 | Current gestational age (from Card) | 1. First trimester 2. Second trimester 3. Third trimester |  |
| 402 | Gestational age at first antenatal care visit (from card) | 1. First trimester 2. Second trimester 3. Third trimester |  |
| 403 | How many times did you get pregnant? | ________________ | If ‘1’ go-to ‘Q407’ |
| 404 | How many total numbers of live births | ________________ |  |
| 405 | Have you ever experienced miscarriage /abortion? | 1. Yes 2. No |  |
| 406 | Have you ever history of still birth | 1. Yes 2. No |  |
| 407 | How many times did you attend this health facility for ANC visit? |  |  |
| 408 | Do the current pregnancy was a planned pregnancy? | 1. Yes 2. No |  |
| 409 | Do you know when you are going to give birth/ Expected Date of Delivery | 1. Yes 2. No |  |
| 410 | How old were you when you got married? (in a completed year) | _____________ |  |
| 411 | How old were you on your first pregnancy? (in a completed year) |  |  |
| 412 | Who make a decision for obstetric service seeking for you? | 1.Self 2. Husband/Relatives 3. Husband/Relatives  4.Me & Husband jointly 5. Others (specify)_____________ |  |
| **Part 5: Maternal level of birth preparedness and complication readiness plan** | | | |
| 501 | What have you done so far to prepare for child birth? **(multiple answer is possible)** | 1. Identify place of delivery 2. Identify skilled provider 3. Save money 4. Identify means of emergency transport 5. Arrange a blood donor for emergency 6. Identify emergency obstetric signs 7. Identify health institution with 24 hours emergency obstetric care 8. Prepare clean clothes & other materials 9. Arrange for emergency fund 10. Make a plan for communication means 11. Identify support people to help 12. Identify the importance of seeking care without delay |  |
